# Correlation of T2-Weighted Magnetic Resonance Imaging (MRI), Computed Tomography (CT), and Cone Beam Computed Tomography (CBCT) Radiomic Features for Prostate Cancer

**DOI:** 10.7759/cureus.80090

**Published:** 2025-03-05

**Authors:** Rodrigo Delgadillo, Benjamin O Spieler, John C Ford, Fei Yang, Matthew Studenski, Kyle R Padgett, Anthony M Deana, William Jin, Matthew C Abramowitz, Alan Dal Pra, Radka Stoyanova, Nesrin Dogan

**Affiliations:** 1 Radiation Oncology, University of Miami Sylvester Comprehensive Cancer Center, Miller School of Medicine, Miami, USA; 2 Radiation Oncology, Varian Medical Systems, Phoenix, USA

**Keywords:** cone beam ct, ct, mri, prostate cancer, radiomics

## Abstract

Radiomics extracted from cone beam computed tomography (CBCT) can be assessed at time points during treatment and may provide an advantage over assessments in a pre-treatment setting using diagnostic images, like magnetic resonance imaging (MRI) or computed tomography (CT), for prostate cancer (pCa) patients receiving radiotherapy (RT). The purpose of this study was to analyze correlations between prostate radiomic features (RFs) derived from T2-weighted (T2w) MRI, CT, and first fraction CBCT for patients receiving RT for pCa. Forty-seven patients were analyzed. The prostate volumes were manually segmented, and 42 radiomic features were extracted, of which seven volume-normalized RFs were considered. The absolute Spearman correlation was calculated among the RFs of the aforementioned imaging modalities (R_M_) and prostate volume (R_V_) since the motivation of this paper was to analyze the strength of the correlation. The Benjamini-Hochberg adjustment was applied to p-values to account for multiple comparisons. No high correlations were found between CT/CBCT vs. T2w. The intramodality R_M_ demonstrated that CT RFs were much higher than the other modalities. For example, intramodality R_M_≥0.95 the percentage of RFs was 17% for CT, 9% for CBCT, and 4.5% for T2w. The differences in RFs across different modalities can be viewed positively: the lack of correlation between RFs across T2w and CT/CBCT could indicate a fundamental difference in the extractable image information. It could also indicate that some RFs did not have any extractable information. A future study will include evaluating the predictive performance of patient outcomes using radiomic features from CT, CBCT, and T2w, which could help in answering such questions.

## Introduction

A variety of radiomics studies to date have proposed that radiomic features (RFs) are predictive for the prognosis and treatment response in prostate cancer (pCa) patients treated with radiotherapy (RT) [1-11], but little is known about the applicability of the same RFs across different imaging modalities. The clinical relevance of understanding correlations across imaging modalities may be to leverage knowledge gained from one modality to another. Alternatively, differences in the RFs may indicate complementary information from the imaging modalities.

However, it should be acknowledged that MRI, CT, and CBCT contain different image information that is likely to yield unequal quantities for the RFs. For example, MRI soft tissue contrast in the prostate is much greater than that of CT. Consequently, image contrast differences between MRI, CT, and CBCT that lead to low radiomic feature correlations may indicate that information content among these modalities is complementary.

This exploratory study investigates RFs extracted from pCa patients receiving RT and scanned with T2-weighted (T2w), CT, and CBCT. Previous publications have warned about potential confounding effects due to the volume dependence of RFs [12-14]. Therefore, we also investigated the volume dependence of RFs for each of these modalities.

The primary objective of this study was to evaluate the correlation of radiomic features (RFs) extracted from T2-weighted MRI, CT, and first-fraction CBCT images of the prostate in patients receiving radiotherapy for prostate cancer. A secondary objective was to assess the volume dependence of these RFs for each imaging modality.

## Materials and methods

Patient population

Forty-seven patients were retrospectively selected from a database of patients that were enrolled in institutional review board (IRB)-approved protocols for the treatment of pCa. The pCa patient population included a variety of Gleason Scores, such as 3+3,4+3,4+4,3+4, and 4+5, and staging, such as T1c, T2a, T2b, T2c, T3a, and T3b. For this study, selection criteria required (1) that patients were treated with volumetric modulated radiation therapy (VMAT) on a TrueBeam linear accelerator (Varian Medical Systems, Palo Alto, California), including an iterative reconstruction algorithm to improve CBCT image quality; and (2) that T2w, CT, and CBCT images were available for analysis. Imaging characteristics and sequences are described in the following section. RT dose may lead to anatomical differences in the CBCT as treatment progresses. Meaning that the first fraction of CBCT images before treatment should have the most similarity to the CT and T2w images. For this reason, the first fraction of the CBCT was included in the analysis. The T2w images were limited to scans acquired within three months of the planning CT (pCT) (20.7 ± 25.9 days prior) to reduce the effect of changing anatomy. Image quality was also assessed for inclusion in the study. Patients with prostheses were very obese, and images with other artifacts limited the quality of images that were removed from inclusion in the study. These selection criteria reduced the number of analyzable patients to 47. The mean patient age was 71± 8 years.

Imaging characteristics

Imaging characteristics considered in this study are listed in Table 1. Characteristics such as image size, pixel size, slice thickness, and field of view (FOV) can affect RFs, and describing them can aid in repeatability for future studies. Patients were scanned on a variety of MR models, summarized in Table 1. Patients were simulated using a variety of CT scanners, summarized in Table 1. Daily setup images of the patients were acquired on TrueBeam with onboard imagers reconstructed with an iterative reconstruction algorithm [15]. Based on previous work, a sharp convolution algorithm and very low noise suppression were used to improve image quality and reproducibility with planning CT [16]. For consistency, all images were resampled using a cubic interpolation to 1 mm isotropic voxel size, which is close to the native resolution.

**Table 1:** Imaging characteristics categorized by imaging modality, manufacturer, and model. The number of patients (N) is shown per category. Values for the imaging characteristics are shown using the following notation: mean ± std. FBP is filtered back projection. Manufacturers included Varian Medical Systems, Palo Alto, CA; Siemens Healthineers AG, Germany; Philips, Netherlands, and GE Medical Systems, Chicago, IL. iCBCT: iterative cone-beam computed tomography, CBCT: Cone-beam computed tomography, CT: Computed tomography

Modality	T2	T2	CT	CT	CBCT
Manufacturer	Siemens	GE	Siemens	Philips	Varian
N	31	16	44	3	47
Width (pixels)	458.3 ± 61.8	320 ± 114.5	512 ± 0	512 ± 0	466 ± 0
Height (pixels)	441.8 ± 70.9	320 ± 114.5	512 ± 0	512 ± 0	466 ± 0
Pixel Spacing (mm)	0.7 ± 0.1	1.1 ± 0.3	1.3 ± 0.2	1 ± 0	1 ± 0
Slice Thickness (mm)	2.8 ± 0.5	2.5 ± 0	2 ± 0	2 ± 0	1 ± 0
Tube Voltage (kVp)	N/A	N/A	120.9 ± 4.2	120 ± 0	126.6 ± 4.7
Tube Current (mA)	N/A	N/A	336 ± 156.3	330 ± 14.8	61.6 ± 4.7
Reconstruction	N/A	N/A	FBP	FBP	iCBCT
Convolution Kernel	N/A	N/A	B30s,B41s,Bf42s,I31f	B	Sharp
iCBCT Noise Suppression	N/A	N/A	N/A	N/A	Very Low
Magnetic Field Strength (T)	3 ± 0	3 ± 0	N/A	N/A	N/A
Sequence	Turbo Spin Echo	Fast Spin Echo	N/A	N/A	N/A
Echo Time (ms)	110.7 ± 7.8	84.6 ± 2.1	N/A	N/A	N/A
Repetition Time (ms)	5655.8 ± 1290.1	5964.5 ± 430.9	N/A	N/A	N/A
Flip Angle (deg)	124.9 ± 12.9	111 ± 0	N/A	N/A	N/A
Pixel Bandwidth (Hz/pixel)	315.3 ± 46	341.8 ± 87.3	N/A	N/A	N/A

RF extraction

Forty-two RFs were extracted from prostate contours from the previously mentioned imaging modalities. Textural parameters were calculated from five RF classes, including gray-level co-occurrence matrices (GLCM), neighborhood gray-tone difference matrix (NGTDM), gray-level run-length matrices (GLRLM), and gray-level size zone matrices (GLSZM), and first-order statistical features. The RFs are listed in Table 2. Data processing and radiomic feature analysis were performed using scientific computation software (MATLAB, ver. 2020b, MathWorks Inc., Natick, MA). Radiomic features were calculated using the MATLAB ‘Radiomics’ package developed by Vallières et al., which is publicly available, to extract 3D bitmaps of the ROI using the digital imaging and communications in medicine (DICOM) structure files from the CBCT, CT, and MRI DICOM files [17]. The extracted images from the previously mentioned imaging modalities were isotropically resampled to a 1 mm voxel size. These features are described in detail in Delgadillo et al., including the image biomarker standardization initiative (IBSI) code equivalent [16,18]. While 40 RFs were IBSI compliant, NGTDM coarseness and strength were not IBSI compliant. However, their definitions can be found in Amadasun and King [19].

Previous studies have noted that some radiomic features are highly correlated with volume [12,20-22]. In order to account for possible volume confounding effects, radiomic features that were found to be volume-dependent were normalized using equations stated in Fave et al. and Shafiq-ul-Hassan et al. [20,21]. Volume normalizations (VN) from Fave et al. were used for NGTDM Busyness and NGTDM Coarseness [20]. For NGTDM Strength, GLSZM GLN, GLRLM GLN, and GLRLM RLN volume normalization from Shafiq-ul-Hassan et al. was used [21]. GLSZM ZSN was normalized by dividing by the number of pixels using a similar logic as GLRLM RLN. Formulas used for volume normalization (Table 2) included:

· $RF_{Norm Type 1}=\frac{RF}{V}$

· $RF_{Norm Type 2}=RF\times V$

· $RF_{Norm Type 3}= \frac{\sum_{i=0}^{G_h}p_i\frac{s(i)}{N(i)}}{\sum_{i=0}^{G_h}\sum_{j=0}^{G_h}ip_i-ip_j}$

· $RF_{Norm Type 4}=\left[ \epsilon+ \sum_{i=0}^{G_h}p_i\frac{s(i)}{N(i)}\right]^{-1}$

**Table 2:** Forty-two radiomic features were considered in this study. IBSI codes listed in brackets. Volume normalization is shown when applicable. GLCM: Gray-level co-occurrence matrices, GLRLM: Gray-level run length matrices, IS: Intensity-based statistics, NGTDM: Neighborhood gray-tone difference matrix, GLZSM: Gray-level zone size matrices, IBSI: Image biomarker standardization initiative

Class	Radiomic Feature	Volume Normalization
Gray-level Co-occurrence Matrices (GLCM) [LFYI]	Contrast [ACUI]	
GLCM[LFYI]	Correlation [NI2N]	
GLCM	Dissimilarity [8S9J]	
GLCM	Energy [8ZQL]	
GLCM	Entropy [TU9B]	
GLCM	Homogeneity [IB1Z]	
GLCM	Sum Average [ZGXS]	
GLCM	Variance [UR99]	
Gray-level Run Length Matrices (GLRLM) [TP0I]	Gray-level Non-Uniformity (GLN) [R5YN]	RF_Norm Type 1_
GLRLM	Gray-level Variance (GLV) [8CE5]	
GLRLM	High Gray-level Run Emphasis (HGRE) [G3QZ]	
GLRLM	Long Run Emphasis (LRE) [W4KF]	
GLRLM	Long Run High Gray-level Emphasis (LRHGE) [3KUM]	
GLRLM	Long Run Low Gray-level Emphasis (LRLGE) [IVPO]	
GLRLM	Low Gray-level Run Emphasis (LGRE) [V3SW]	
GLRLM	Run Length Variance (RLV) [SXLW]	
GLRLM	Run Percentage (RP) [9ZK5]	
GLRLM	Run-Length Non-Uniformity (RLN) [W92Y]	RF_Norm Type 1_
GLRLM	Short Run Emphasis (SRE) [220V]	
GLRLM	Short Run High Gray-level Emphasis (SRHGE) [GD3A]	
GLRLM	Short Run Low Gray-level Emphasis (SRLGE) [HTZT]	
Intensity-based Statistics (IS) [UHIW]	Kurtosis [IPH6]	
IS	Skewness [KE2A]	
IS	Variance [ECT3]	
Neighborhood Gray-Tone Difference Matrix (NGTDM) [IPET]	Busyness (BUSY) [NQ30]	RF_Norm Type 3_
NGTDM	Coarseness (COAR)*	RF_Norm Type 4_
NGTDM	Complexity (CPLX) [HDEZ]	
NGTDM	Contrast (CONT) [65HE]	
NGTDM	Strength (STRG)*	RF_Norm Type 2_
Gray-level Zone Size Matrices (GLZSM) [9SAK]	Gray-level Non-Uniformity (GLN) [JNSA]	RF_Norm Type 1_
GLZSM	Gray-level Variance (GLV) [BYLV]	
GLZSM	High Gray-level Zone Emphasis (HGZE) [5GN9]	
GLZSM	Large Zone Emphasis (LZE) [48P8]	
GLZSM	Large Zones High Gray-level Emphasis (LZHGE) [J17V]	
GLZSM	Large Zones Low Gray-level Emphasis (LZLGE) [YH51]	
GLZSM	Low Gray-level Zone Emphasis (LGZE) [XMSY]	
GLZSM	Short Zone Emphasis (SZE) [5QRC]	
GLZSM	Short Zones High Gray-level Emphasis (SZHGE) [HW1V]	
GLZSM	Short Zones Low Gray-level Emphasis (SZLGE) [5RAI]	
GLZSM	Zone Percentage (ZP) [P30P]	
GLZSM	Zone Size Non-Uniformity (ZSN) [4JP3]	RF_Norm Type 1_
GLZSM	Zone Size Variance (ZSV) [3NSA]	

Terms in volume normalization include the number of voxels of each intensity, i, in the image N(i); Nv is the total number of voxels; pi is the probability of intensity i in the image; s(i) is the sum of the average difference value around voxels of intensity i; Gh is the highest gray-level intensity; Ng is the number of gray levels.

Prior to RF extraction, a variety of preprocessing steps can be carried out, including different quantization algorithms, quantization bins, and the use of Collewet normalization [16]. For this work, we focused on the Lloyd quantization algorithm with Collewet normalization since it is highly reproducible [16] between CT and CBCT with preprocessing settings commonly used in MRI RF studies [23]. The IBSI notation refers to this type of Collewet normalization as a re-segmentation method RS:3σ 7ACA [18], and it was calculated by normalizing the gray levels of the ROI from the range of where was the mean and was the standard deviation of the ROI gray levels. The Lloyd-Max quantization algorithm is an algorithm where bin levels are assigned in a way that minimizes quantization error [24]. Sixty-four quantization bins were used. Prostate volume was also included in the RF list to assess its impact on other RFs.

Prostate delineation

The standard clinical workflow for contouring the prostate follows the Prostate Imaging Reporting and Data System (PIRADS [25]). The entire prostate, including the urethra, was segmented for radiomic analysis. A team of radiation oncologists with expertise in prostate cancer RT delineated the prostate on the planning CT (pCT) with the aid of MRI. The prostate volumes were segmented using commercial radiation oncology imaging software that included MIM, ver. 6.8.1, MIM Software Inc., Cleveland, OH, and Eclipse, ver. 16.1, Varian Medical Systems, Inc., Palo Alto, CA. The prostate contours were drawn by the radiation oncologist on either the MRI or pCT, then rigidly transferred to the other. The CBCT prostate contour was rigidly transferred from the pCT prostate contour. When necessary, the radiation oncologist corrected the prostate contours to account for prostate deformation or contour artifacts due to registration errors. Gold fiducial artifacts were removed from prostate contours on CT and CBCT images prior to RF extraction using the algorithm described in a previous study [16]. The fiducial artifact removal algorithm sets an artifact threshold defined with range [μAL − 3σAL, μAL + 3σAL] where μAL is the mean and σAL is the standard deviation of the voxel intensity levels on layers not containing fiducial artifacts. On layers containing fiducial artifacts, a mask was generated by defining a circle of 5 mm centered on the fiducial marker, including pixels that exceeded the artifacts threshold. Typically, metal streaking artifacts in the prostate radiate outwards from the center of the fiducial. To capture this aspect, masks were generated to overlay pixel mask lines between the distal artifact pixels to the center of the fiducial [16]. For this study, the removal of the gold fiducial artifacts reduced the ROI volumes of the CT by 15 ± 13% (average ± standard deviation) and the CBCT by 10 ± 9%. Gold fiducial artifacts were not removed from the T2w ROI as streaking from gold fiducial artifacts was not present on T2w as it is in CT. For a subsample of 10 patients, the CT gold fiducial artifact mask was manually recreated in MIM imaging software and rigidly transferred to the T2 images. Then, the radiomic features of the prostate on the T2 images were calculated both with and without the artifact mask applied. A paired T-test was performed between the RFs with and without the artifacts mask applied to the T2w images.

Data analysis

**Figure 1:**
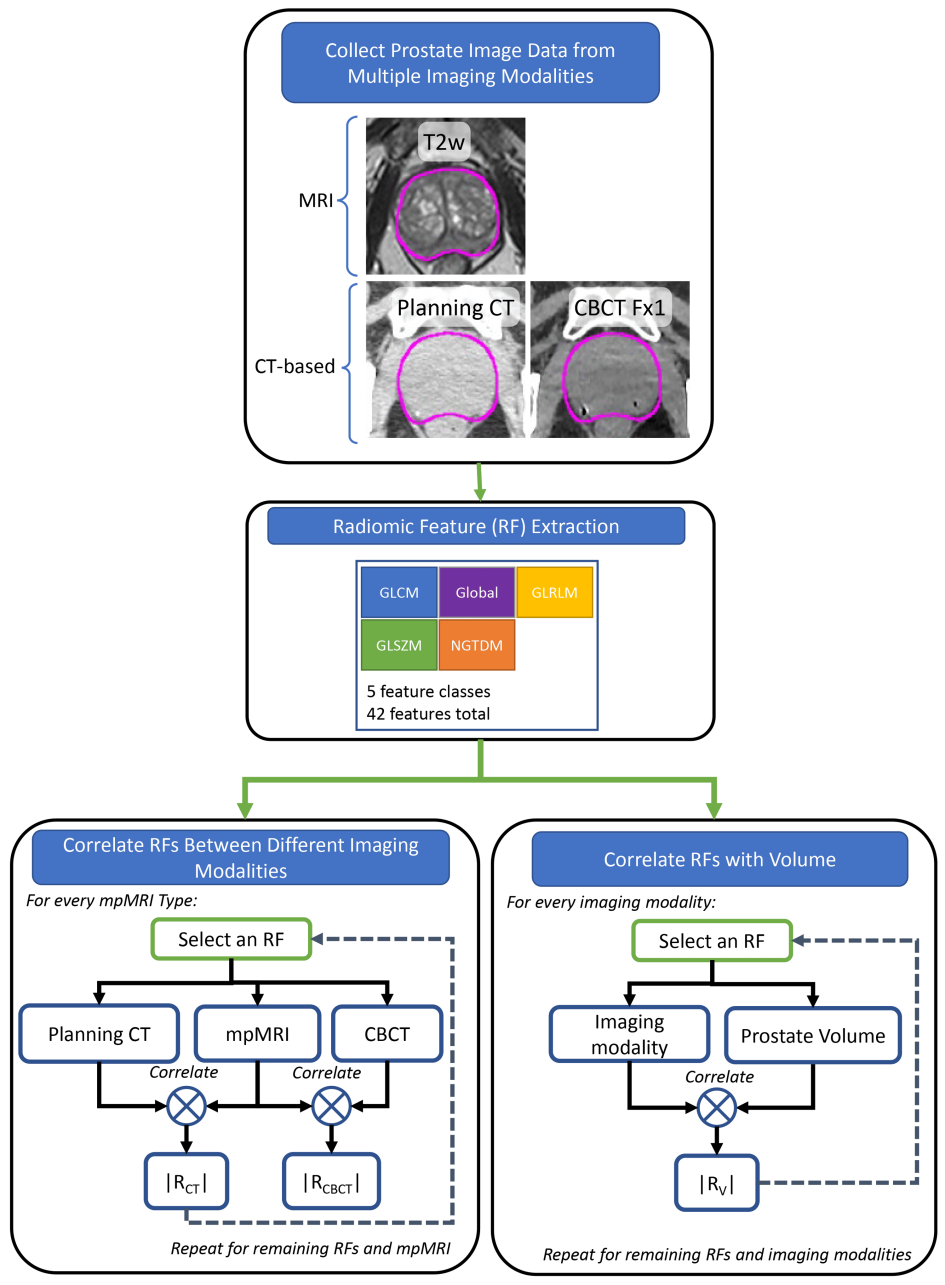
Workflow for estimating the correlation of RFs between T2w, CT, and CBCT, and RFs vs. prostate volume for each imaging modality. CT: Computed Tomography, CBCT: Cone Beam Computed Tomography, RF: Radiomic Features

To investigate associations between imaging modalities, the absolute value of the Spearman correlation coefficient (R) was calculated between the RF from T2w and the same RF from CT/CBCT. The absolute value of R was considered because both strongly positive and negative correlations indicate an association between variables. The workflow for this data analysis is shown in Figure 1. Spearman correlation was utilized to account for non-normal distributions. The threshold for well-correlated RF was R>0.75. Data processing and analysis were performed using scientific computation software (MATLAB). The Benjamini-Hochberg correction was used to adjust the p-values to account for multiple comparisons [26]. Precedence for using the Benjamini-Hochberg for multiple comparisons was found in a lung-based radiomics paper by Fave et al. 2016 [20]. An alpha of 0.05 was chosen to set the significance threshold for the adjusted p-value. To further study the role of volume on the RFs, the correlation between prostate volume and RF was calculated for every RF and imaging modality.

A correlation matrix was calculated between all combinations of RFs and modalities considered in this study. The diagonal components of the correlation matrix are trivially equal to one and removed from the analysis. The correlation matrix is symmetric about the diagonal, and counting all entries would lead to double counts. Only the triangular upper part of the p-values matrix was considered in the Benjamini-Hochberg correction. RFs with a correlation greater than 0.75 with volume were highly removed from the analysis to avoid volume confounding effects, which is consistent with our threshold for well-correlated as stated earlier.

Two types of correlation comparisons were considered for the analysis.

The first type of correlation comparison was intramodality correlations of RFs. These are correlations within the same modality but across different RFs. It could be that modalities exhibit differences in the self-correlations within the same modality. Some modalities may tend to have a smaller effective feature space than others if many of their RFs correlate with each other. Future work could assess the effective feature argument by exploring a principal component analysis, or similar technique, to assess the dimensionality of the feature space in each modality.

The second type of correlation comparison was the intermodality correlations of RFs. These are correlations between different modalities. These types of correlations are studied to analyze whether RFs are robust across modalities. There are two types of intermodality correlations of RFs considered in this study. The first type of intermodality correlations was the same RF comparisons only. It could be that a particular RF from one modality correlates with the same RF on another modality. For example, CT and CBCT are more similar to each other than T2w. So, it may be expected that more RFs are robust between CT vs. CBCT than CT vs. T2w. The second type of intermodality correlation was variable modality-RF comparisons. When looking at T2w, it is often noticed that some areas that appear high intensity in T2w appear low intensity in CT or vice versa. So, it may be possible that an RF from T2w may compare to a different RF from CT, for example. Perhaps a feature counting segments of low intensities correlates to another feature counting segments of high intensity on a different modality.

## Results

The effect of artifact mask on radiomic features

A paired T-test showed that most of the RFs were not statistically different with and without the artifacts mask applied to the T2w images, shown in Table 3. However, a few were statistically significant (p<0.05) and were all from the size zone and run length matrices, which makes intuitive sense because the streaking artifacts introduce breaks in the continuous runs of the same gray levels.

**Table 3:** Statistical comparison between radiomics features extracted from T2 images with and without an artifact mask. GLCM: Gray-level co-occurrence matrices, RF: Radiomic features, GLN-VN: Gray-level non-uniformity VN, GLRLM: Gray-level run length matrices, GLV: Gray-level Variance, HGRE: High gray-level run emphasis, LGRE: Low gray-level run emphasis, LRE: Long run emphasis, LRHGE: Long run high gray-level emphasis, LRLGE: Long run low gray-level emphasis, RLN-VN: Run-length non-uniformity (VN), RLV: Run-length non-uniformity, RP: Run percentage, SRE: Short run emphasis, SRHGE: Short run high gray-level emphasis, SRLGE: Short run low gray-level emphasis, GLSZM: Gray-level size zone matrices, HGZE: High gray-level zone emphasis, LGZE: Low Gray-level zone emphasis, LZE: Large zone emphasis, LZHGE: Large zones high gray-level emphasis, LZLGE: Large zones low gray-level emphasis, SZE: Short zone emphasis, SZHGE: Short zones high gray-level emphasis, SZLGE: Short zones low gray-level emphasis, ZP: Zone percentage, ZSV: Zone size variance, NGTDM: Neighborhood gray-tone difference matrix

		Spearman Correlation		Paired T-test	
Feature Class	RF	r	p-value	t	p-value
GLCM	Contrast	0.794	0.010	-1.924	0.087
GLCM	Correlation	0.964	0.000	0.754	0.470
GLCM	Dissimilarity	0.952	0.000	-2.037	0.072
GLCM	Energy	0.927	0.000	1.399	0.195
GLCM	Entropy	0.903	0.001	-1.514	0.164
GLCM	Homogeneity	0.976	0.000	2.271	0.049
GLCM	SumAverage	1.000	0.000	-0.205	0.842
GLCM	Variance	0.988	0.000	-0.899	0.392
Global	Kurtosis	0.927	0.000	1.728	0.118
Global	Skewness	0.988	0.000	0.406	0.694
Global	Variance	0.976	0.000	-1.366	0.205
GLRLM	GLN-VN	0.939	0.000	1.820	0.102
GLRLM	GLV	0.903	0.001	1.303	0.225
GLRLM	HGRE	0.988	0.000	0.270	0.793
GLRLM	LGRE	0.988	0.000	-0.168	0.870
GLRLM	LRE	0.770	0.014	-2.150	0.060
GLRLM	LRHGE	0.964	0.000	-2.189	0.056
GLRLM	LRLGE	0.988	0.000	-0.456	0.659
GLRLM	RLN-VN	0.770	0.014	2.098	0.065
GLRLM	RLV	0.588	0.080	1.184	0.267
GLRLM	RP	0.770	0.014	2.138	0.061
GLRLM	SRE	0.770	0.014	2.178	0.057
GLRLM	SRHGE	0.988	0.000	0.575	0.579
GLRLM	SRLGE	0.988	0.000	-0.037	0.971
GLSZM	GLN-VN	0.782	0.012	2.006	0.076
GLSZM	GLV	0.709	0.028	-0.475	0.646
GLSZM	HGZE	0.988	0.000	-0.210	0.838
GLSZM	LGZE	0.939	0.000	-0.533	0.607
GLSZM	LZE	0.636	0.054	-1.363	0.206
GLSZM	LZHGE	0.515	0.133	-1.138	0.285
GLSZM	LZLGE	0.806	0.008	-2.279	0.049
GLSZM	SZE	0.564	0.096	1.274	0.235
GLSZM	SZHGE	0.927	0.000	0.351	0.734
GLSZM	SZLGE	0.636	0.054	-0.206	0.842
GLSZM	ZP	0.721	0.024	2.040	0.072
GLSZM	ZSV	0.794	0.010	-1.676	0.128
NGTDM	Busyness-VN	0.988	0.000	0.215	0.834
NGTDM	Coarseness-VN	0.964	0.000	0.875	0.405
NGTDM	Complexity	0.733	0.021	-1.676	0.128
NGTDM	Contrast	0.964	0.000	-0.585	0.573
NGTDM	Strength-VN	0.927	0.000	-1.399	0.195
Geometry	Volume	0.709	0.028	-3.002	0.015

Correlation of RFs

**Figure 2:**
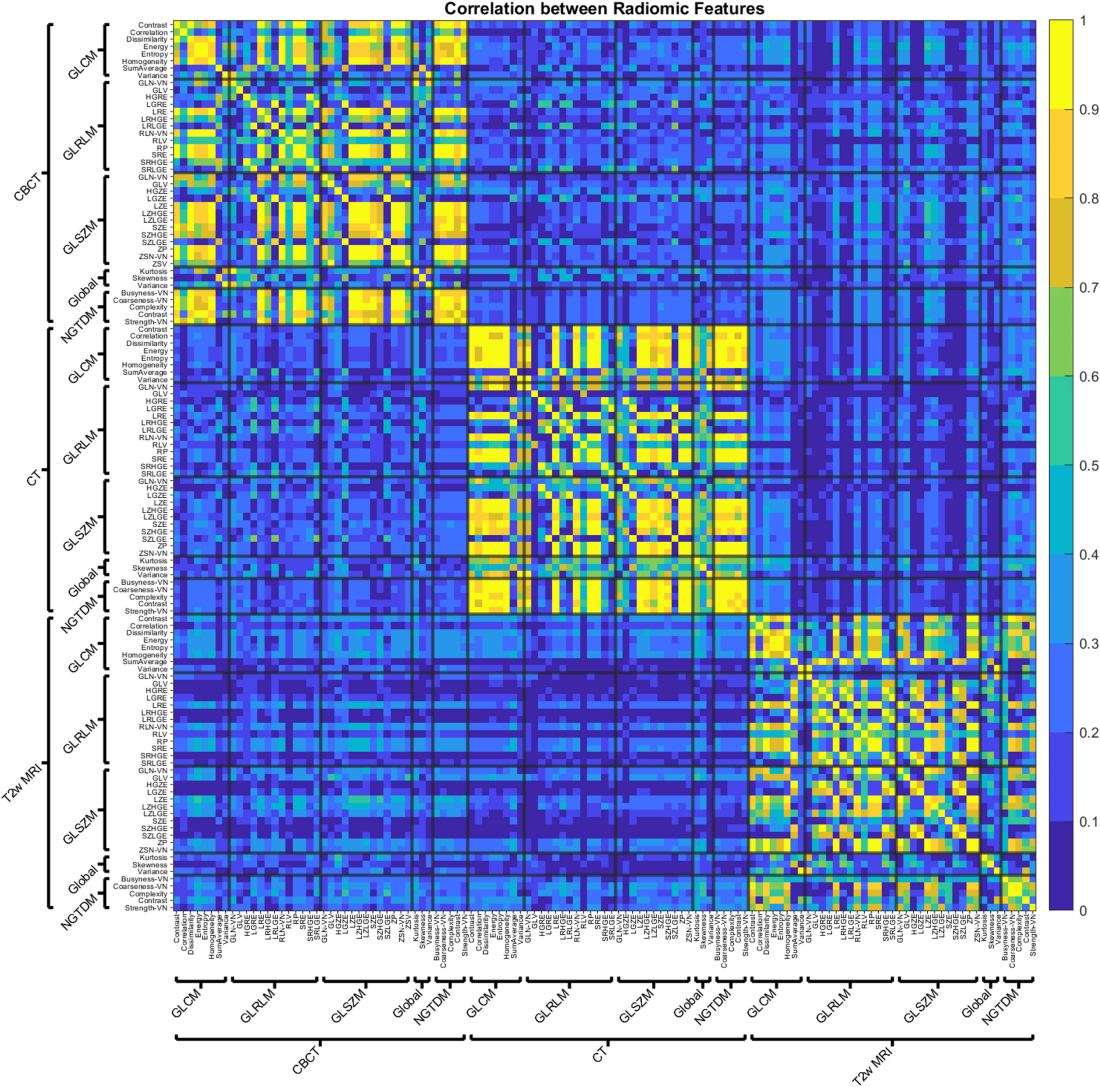
Initial correlation matrix of all the combinations of RFs and modalities considered in this study. Only the upper triangular part of the correlation matrix was considered for the statistical analysis to avoid overcounting and trivial comparisons.

**Figure 3:**
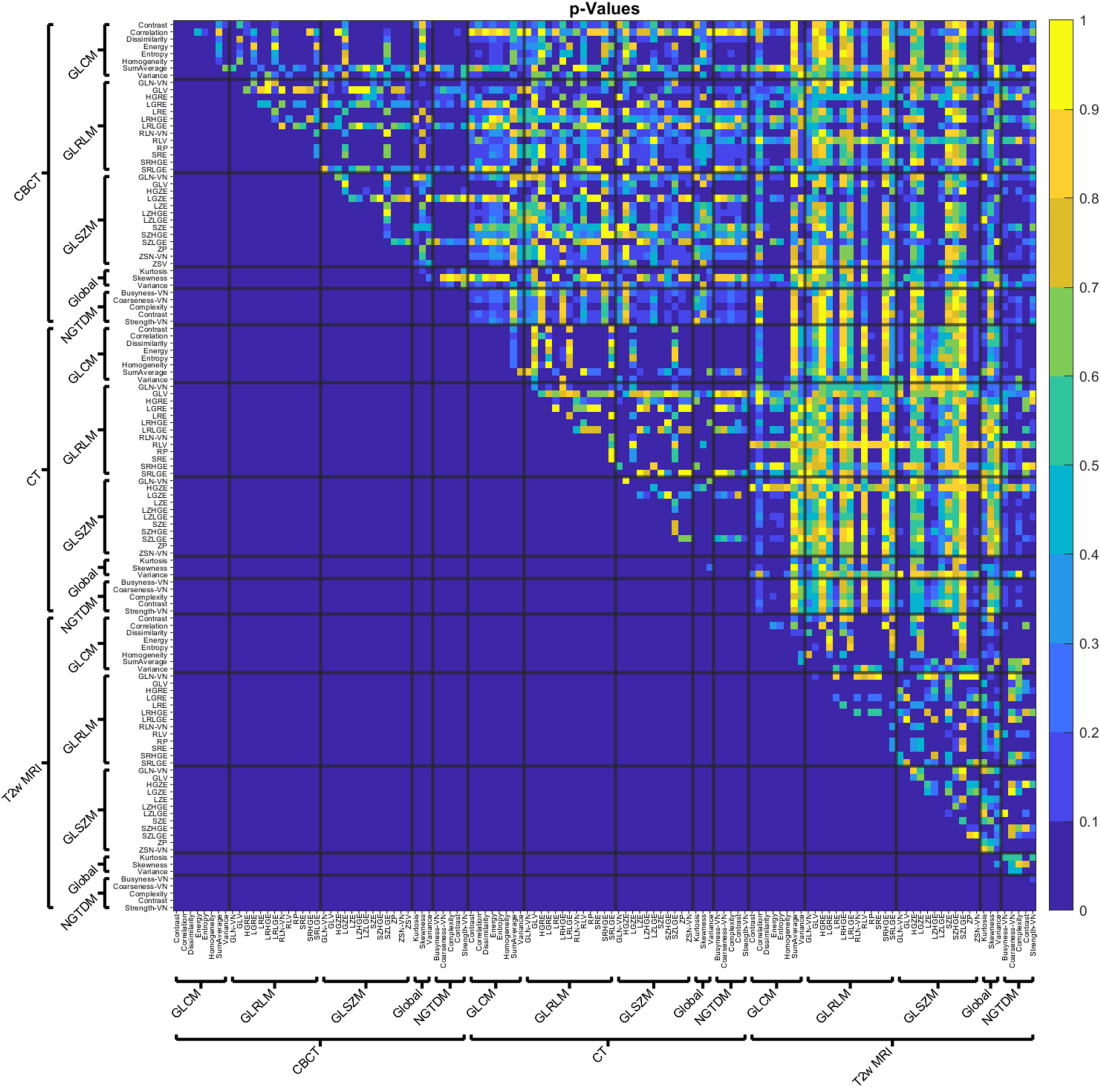
Adjusted p matrix for the correlations considered in this study.

The initial correlation matrix of RFs across different modalities is shown in Figure 2. As described in the methods, only the upper triangular part of the correlation matrix was considered for this study. The adjusted p-values are shown in Figure 3. In contrast to Figure 2, where yellow represents a high correlation, the data in Figure 3 (yellow) corresponds to a high p-value. Some of the differences between the comparisons can be noticed in Figures 2, 3. For example, the intramodality correlations stand out as having higher correlations than the intermodality correlations. Notice the number of higher correlations in the squares along the diagonals in Figure 2. It can also be seen that CT/CBCT vs. T2w correlations often have higher p-values and indicate that the many RF correlations across CT/CBCT vs. T2w are not statistically significant.

**Figure 4:**
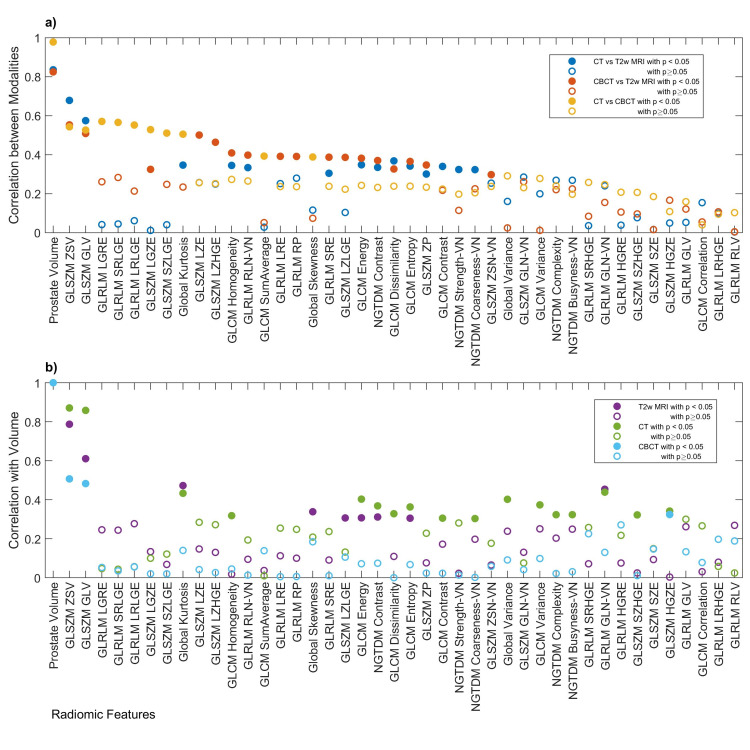
a) Intermodality correlation of RF, RM, vs. RF. Only the same RF comparisons are shown for brevity. b) RF correlation with volume, RV,vs. RF. Values sorted based on max |RM| across all the different modalities in descending order. Redundant RF with pM>0.05 across all modality comparisons was removed from the figure.

The intermodality correlation of RFs and the RF correlation with volume is shown in Figure 4. As stated in the methods, the volume correlations were used to filter out RFs that correlate highly with volume. All the volume-normalized features have intermodality correlations less than 0.5.

Intramodality correlations

**Figure 5:**
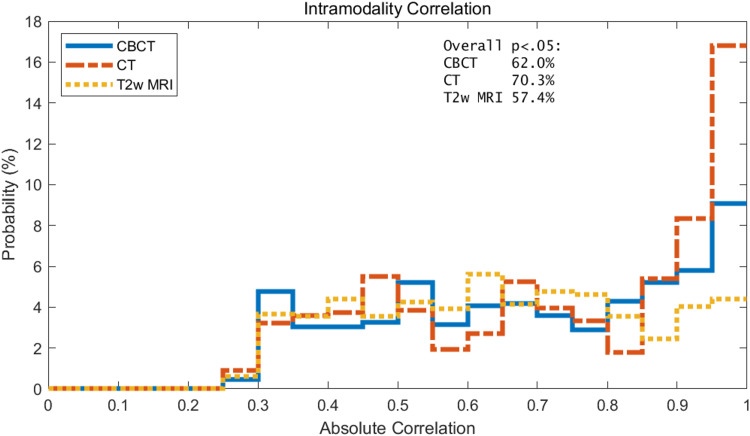
Histogram of the intramodality correlation of RFs for CBCT, CT, and T2w of the prostate. Results shown only include the statistically significant data, i.e., p<0.05. Overall percentages of features that were statistically significant by modality are shown in text box in upper right corner.

A histogram of the intramodality correlations of RFs for CBCT, CT, and T2w of the prostate is shown in Figure 5. CT RFs have the greatest portion of RFs with intramodality correlation greater than 0.85. This is followed by CBCT with the second largest portion of RFs with intramodality correlation greater than 0.85, and T2w is last. As shown in Figure 5, the RFs of T2w are evenly distributed over the intramodality correlations.

Intermodality correlations: same RF comparisons only

**Figure 6:**
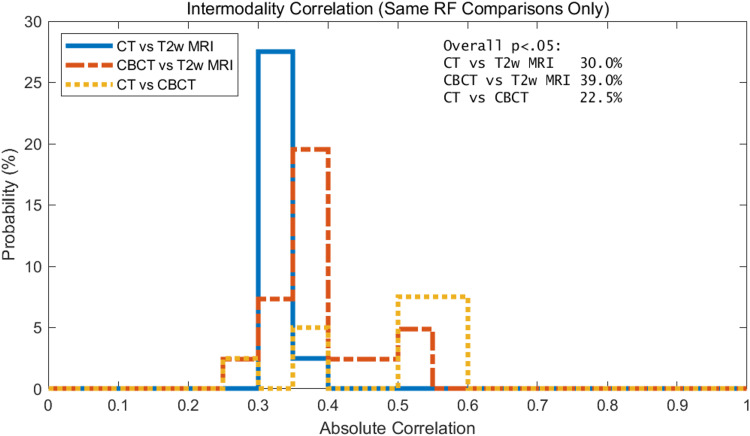
Histogram of the intramodality correlation of RFs for CBCT, CT, and T2w of the prostate. Results shown only include the statistically significant data, i.e., p<0.05. Overall percentages of features that were statistically significant by modality are shown in text box in upper right corner.

When considering the same RFs, CT vs. CBCT RFs correlate more than the CT/CBCT vs. T2w RFs. The intermodality correlation for the same RF comparisons is only shown in Figure 6. As mentioned in the introduction, this is expected since CT and CBCT are both X-ray-based imaging. This makes sense intuitively, a T2w image of the prostate looks different than a CT or CBCT. Figure 6 also demonstrates that more CBCT RFs correlate with T2w than CT RFs with T2 MRI. It can also be noticed that modes of the CT/CBCT vs. T2w RF intermodality correlations are smaller.

Intermodality correlations: variable modality-RF comparisons

**Figure 7:**
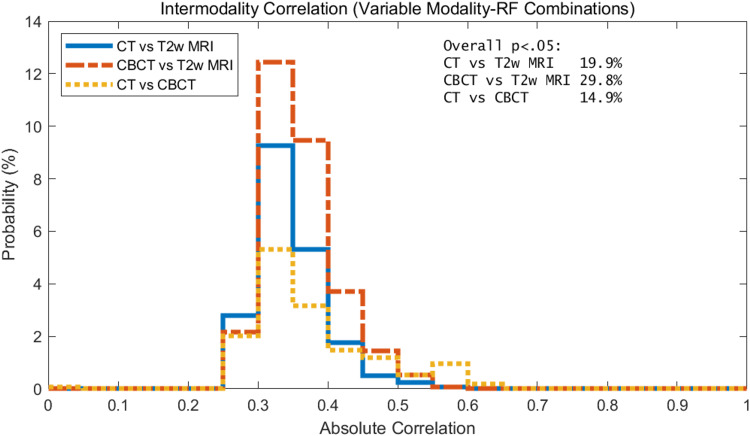
Histogram of the intermodality correlation of RFs for CBCT, CT, and T2w of the prostate for variable modality-RF combinations. Results shown only include the statistically significant data, i.e., p<0.05. Overall percentages of features that were statistically significant by modality are shown in text box in upper right corner.

The intermodality correlation for variable modality-RF combinations is shown in Figure 7. The results show that the mode of the intermodality correlation with variable modality-RF combinations is less than 0.35. Thus, the situation where one RF on T2w correlates highly with a different RF on CT/CBCT was not observed.

## Discussion

In the literature, many radiomics studies of pCa have focused on one modality at a time, such as computed tomography (CT), positron emission tomography (PET), or magnetic resonance imaging (MRI) [5,27]. Also, there have been many radiomics studies of pCa using multiparametric MRI sequences [1-4,6-11]. Previous work has explored the reproducibility of RFs across planning CT (pCT) and first fraction CBCT [16]. That work showed that correlations of RFs from CBCT and pCT RFs depended on the reconstruction algorithm and preprocessing methods used for feature extraction. However, to our knowledge, no previous work has documented the correlation of RFs between MRI, CT, and cone beam CT (CBCT).

As expected, the intermodality correlations of RFs were stronger for CT vs. CBCT than they were for CT vs. T2w or CBCT vs. T2w. The intermodality correlations of RFs had modes less than 0.35 for CT/CBCT vs. T2 MRI, while the mode of the RF correlations between CT and CBCT was around 0.55. The correlation of RFs between CT and CBCT makes sense since those imaging modalities have more in common than T2w.

Another aspect of this work was the analysis of the intramodality comparisons of RFs. It was found that CT RFs often correlate more within the same modality than the other modalities considered. Several interpretations come to mind. First, it could be a result of the known stability of CT. CTs are implemented in the radiotherapy clinic for their small geometrical distortion and high Hounsfield unit reproducibility which is vital for dose calculation algorithms. In our study, there is less imaging modality variation in the X-ray-based machine than in the MRI machines. All planning CT images are located inside our clinic and have imaging protocols tightly controlled by the radiation oncology team. Moreover, many of the CT images used in this study are either similar models or from the same vendor. Similarly, the CBCT image protocols were tightly controlled. However, diagnostic MRIs are often done in another clinic, and hence there is more machine variability. It is also known that T2w signal intensity is highly variable. The same patient, same machine, and same day can have variable intensity due to a variety of factors, including radiofrequency coil placement, pre-scan settings, and receiver gain.

An alternative interpretation is that MRI RF may not correlate with each other because the RFs really are different from each other. T2w is used in prostate cancer radiotherapy because it provides high anatomical detail. It plays a vital role in delineating abnormalities within the prostate. High RF intramodality correlation could indicate a small effective feature space, since it could be argued that if many RFs correlate with each other, then they provide no added information. Thus, it may be that the T2w has a larger effective RF space and is more useful for radiomic studies of prostate cancer.

In pre-study analysis, it was observed that apparent correlations between modalities for several RFs were lost after volume normalization. It was also found that prostate volume correlates well across different imaging modalities. Previous work showed that eight RFs correlated significantly between CT and CBCT using an iterative reconstruction algorithm with a sharp convolution filter and very low noise suppression [16]. The same reconstruction settings were used in this current work for consistency. The eight RFs found to correlate between CT and CBCT were GLRLM RLN, GLRLM GLN, GLSZM GLN, NGTDM Busyness, GLSZM ZSN, NGTDM Coarseness, GLSZM ZSV, and GLSZM GLV [16].

The volume dependence of RFs has been documented previously, and the volume normalization described by Fave et al. [20] and Shafiq-ul-Hassan et al. was used in this work [20-22]. However, neither Fave et al. nor Shafiq-ul-Hassan et al. mentions volume normalization for GLSZM ZSN, and none was found in the literature. A previous radiomics study of breast cancer analyzing T1-weighted and T2w-weighted also documented the volume dependence of GLSZM ZSN [28]. There has been interest in the repeatability of radiomics studies, and it was noticed that many RFs not only correlate with volume but also with other RFs. In a study that analyzed 84 RFs from publicly available datasets of head, neck, and lung cancer, Traverso et al. [12] found that 80% of RFs correlated with other RFs considered in the library, and 30% of RFs correlated with volume. That study suggested RF pairwise correlations and volume correlations before constructing machine learning algorithms based on RFs. An interesting finding in our study was that the volume dependence of RFs differs between imaging modalities. The volume dependency of these features could be related to inflammation in the prostate and extracellular fluid space [29]. In future radiomics studies, failure to account for RF volume dependency could lead to misleading results.

This study has several limitations, including variable timing between the imaging scans, variability in imaging parameters, the presence of imaging artifacts due to gold fiducials, the limited library of radiomic features, and the limited sample size. To improve our understanding, the limitations of this study are further elaborated in the following paragraphs.

The timing between T2w and CT/CBCT was variable. Ideally, they should be acquired on the same day with similar rectum filling, bowel filling, and patient setup. Also, tighter control of image acquisition parameters and the machines used for the MRI and CT would be desirable but can be difficult to control in clinical studies [30,31]. In contrast, CBCT imaging parameters were tightly controlled in this study by virtue of all patients receiving RT on the same linear accelerator with iterative CBCT imaging.

Another limitation was the presence of artifacts from gold fiducials in the prostate, which was mitigated by using an algorithm to remove fiducial artifacts from the prostate contours. However, the fiducials have utility for accurate patient setup and are frequently encountered in patients receiving external beam radiotherapy for prostate cancer. Tighter patient selection criteria, such as limiting the MR and CT scanners allowed or reducing the time interval between the scans of the different modalities, would reduce cohort size below a threshold reasonable for analysis. There are many more RFs that could be considered from other radiomics software libraries. However, the exhaustive inclusion of all RFs was beyond the scope of this exploratory study for several reasons, including the small sample size and the recognition that many radiomic features cross-correlate with one another and thus do not necessarily provide new information [12].

The sample size limitations for this study are more apparent for the lower correlated RFs. For example, let us consider a correlation power analysis using the point biserial model, two tails, and adjusting our alpha of 0.05 to 4.0e-4 using the Bonferroni correction for multiple comparisons. For our sample size of 47 patients, for a large effect of 0.75, the statistical power is 1.00, and for a small effect of 0.3, the statistical power is 0.28. In this study, the correlation values for the highly correlated RFs were around 0.75, and the lower correlated RFs were around 0.3, which is consistent with our observations. The Bonferroni correction is known to minimize false positives in multiple comparisons but is overly harsh and probably produces a false negative [32,33]. However, in this study, we used the Benjamini-Hochberg adjustment, which is less harsh than the Bonferroni correction [20,26]. Our effective alpha is likely slightly higher, but in this statistical power calculation, we present a more conservative approach to be safe. With the limitations of sample size in this study, some of the smaller correlations may be rejected, though they are real. A larger sample size would be needed to study these. Using the same conventions previously discussed, a power analysis indicates that 93 patients are needed to achieve a power of 0.75 for an effect size of 0.3. For the current sample size, alpha level, and desired statistical power of 0.75, the minimum detectable effect size is 0.41. Consequently, a larger sample size would be needed to detect smaller correlations. It should be noted that if we were to extend the sample size of the study, there is still a possibility of detecting weak correlations. The objective of this study was to identify large RF correlations. Thus, the sample size should be sufficient for the purpose of this study.

## Conclusions

T2w, CT, and CBCT imaging modalities are quite different in terms of image quality and contrast. For example, the fatty soft tissue in the prostate is easier to observe on T2w than CT. Moreover, the transition and peripheral zones are difficult to delineate on CT alone, but on MRI they are very noticeable. Given all these differences, one would expect quantitative textural information to also be different. For future studies, these differences can be viewed as complementary: the lack of correlation between RFs across T2w and CT/CBCT could indicate a fundamental difference in the extractable image information. The specific RFs investigated in this study did not demonstrate strong correlations across modalities. However, there is an open possibility that other RFs or analysis methods may reveal meaningful relationships. A future study could be to evaluate the predictive performance of patient outcomes using radiomic features from CT, CBCT, and T2w to further ascertain whether the imaging modalities are complementary. If the information is non-complimentary, one might as well just rely on one modality since that is a more efficient process. However, if the multimodal features are complementary information may enhance predictive radiomic models of prostate cancer by combining the best features from each modality.
